# Supplementary figures and images for: Early recovery of proteasome activity in cells pulse-treated with proteasome inhibitors is independent of DDI2
Source: eLife. 2024 Apr 15;12:RP91678. doi: 10.7554/eLife.91678 (PMC11018354; doi:10.7554/eLife.91678)

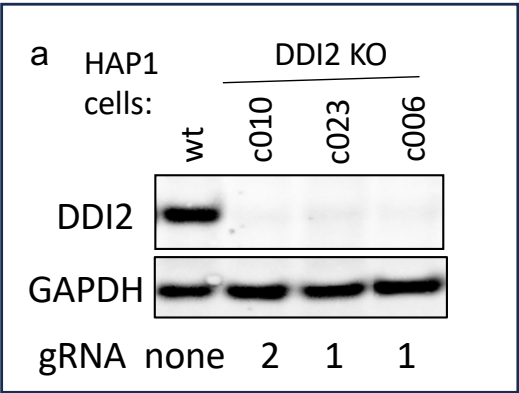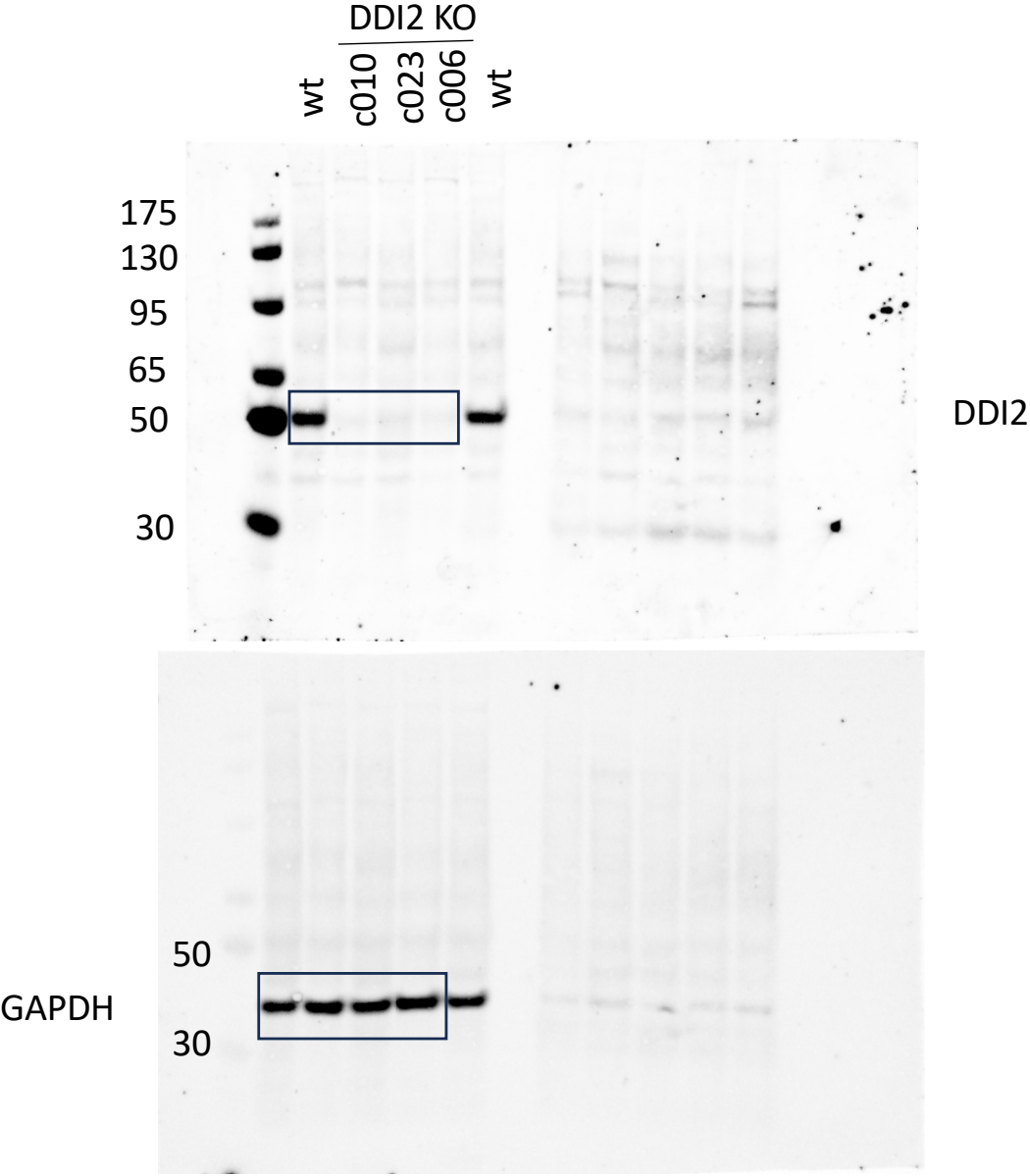

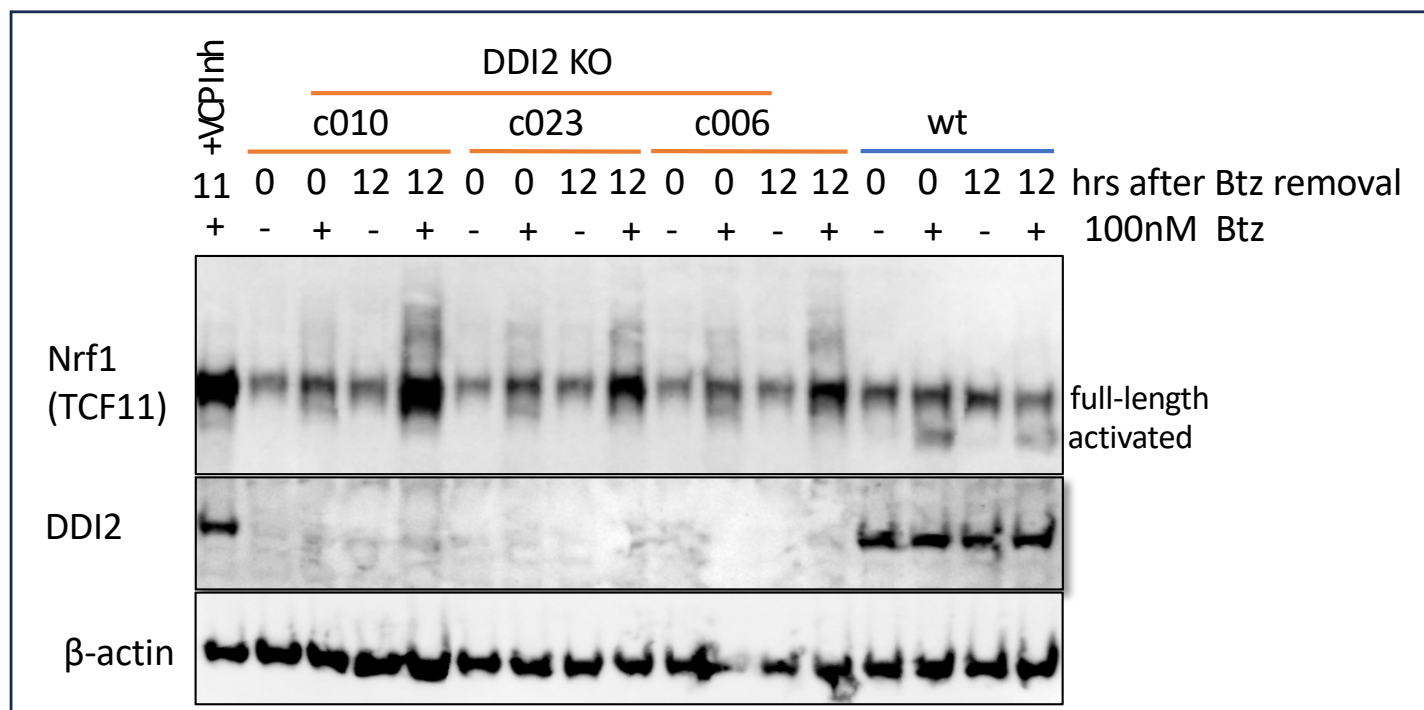

Nrf1 (TCF11)

DDI2 Ab after Nrf1 antibodies

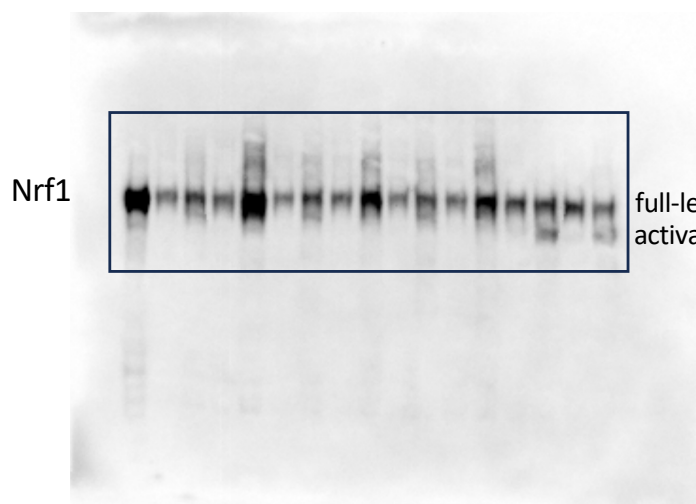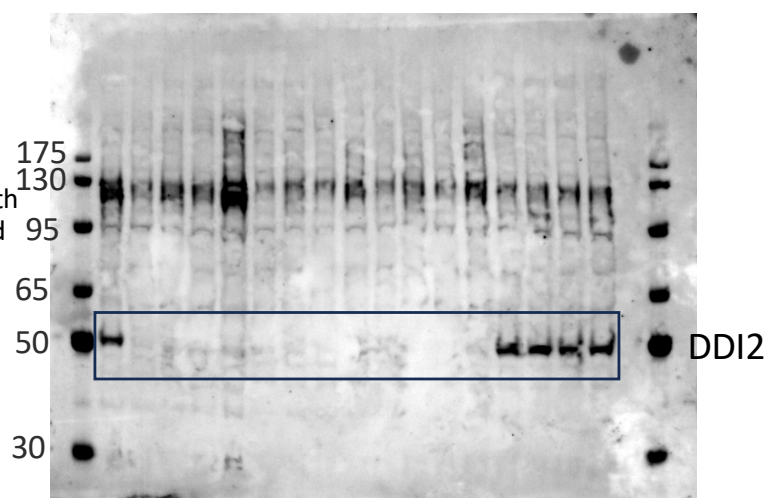

β-actin

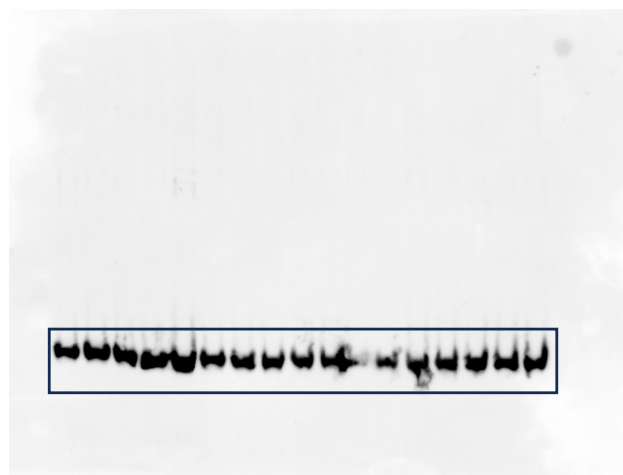

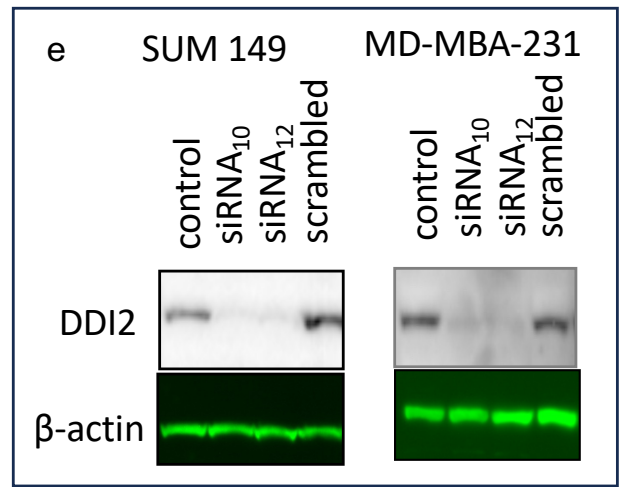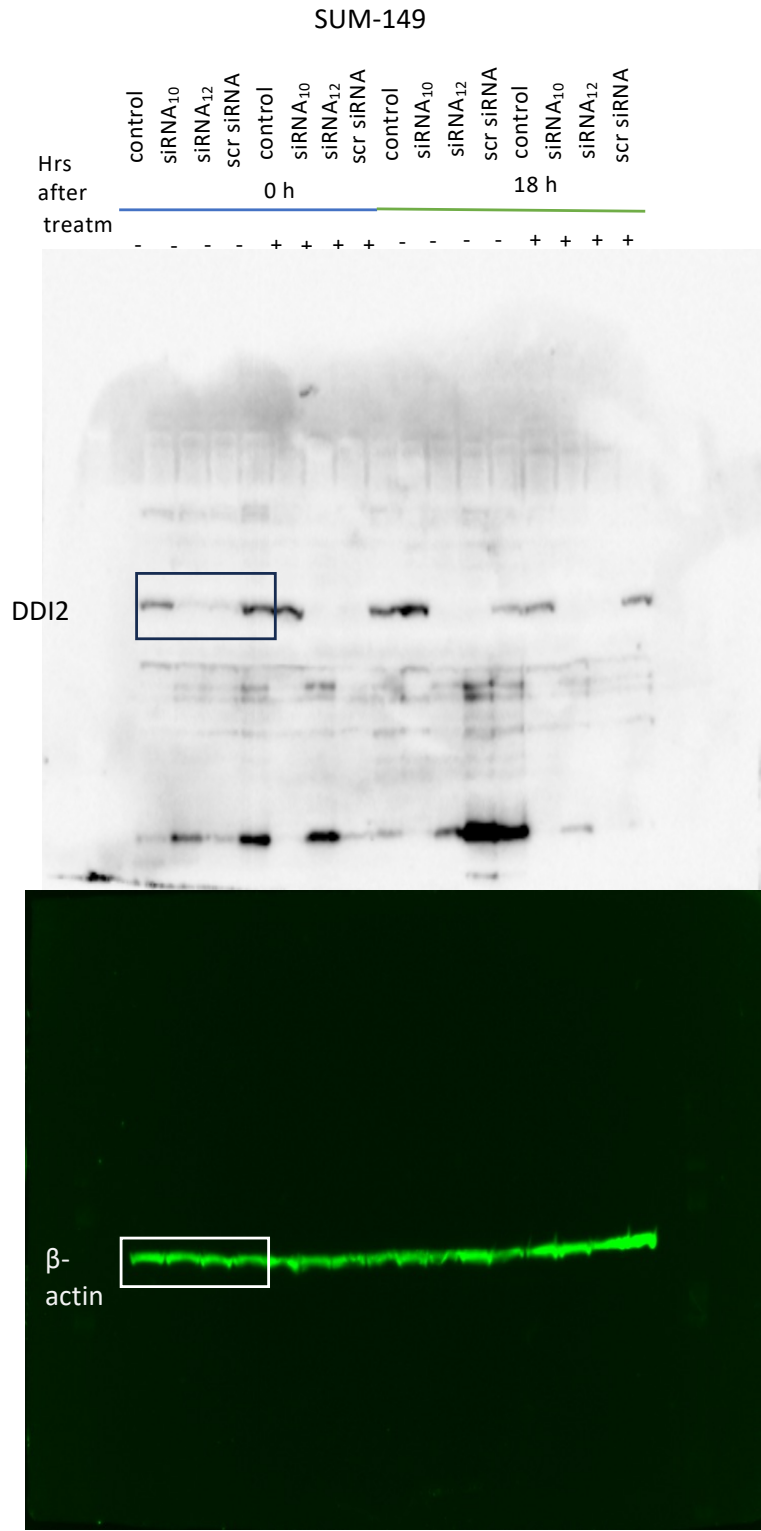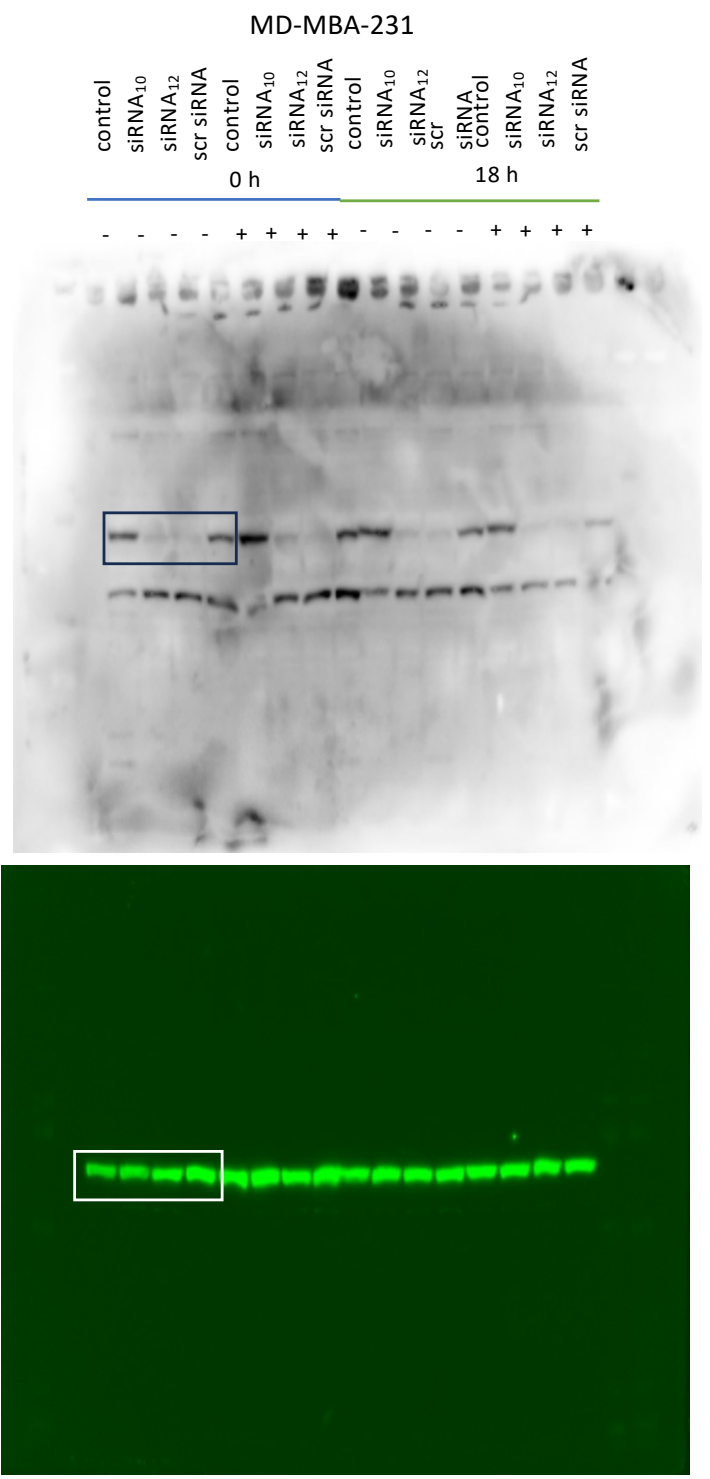

Supplement: Figure 1—source data 1. [file elife-91678-fig1-data1.zip › Fid1_source1 corrected.pdf]

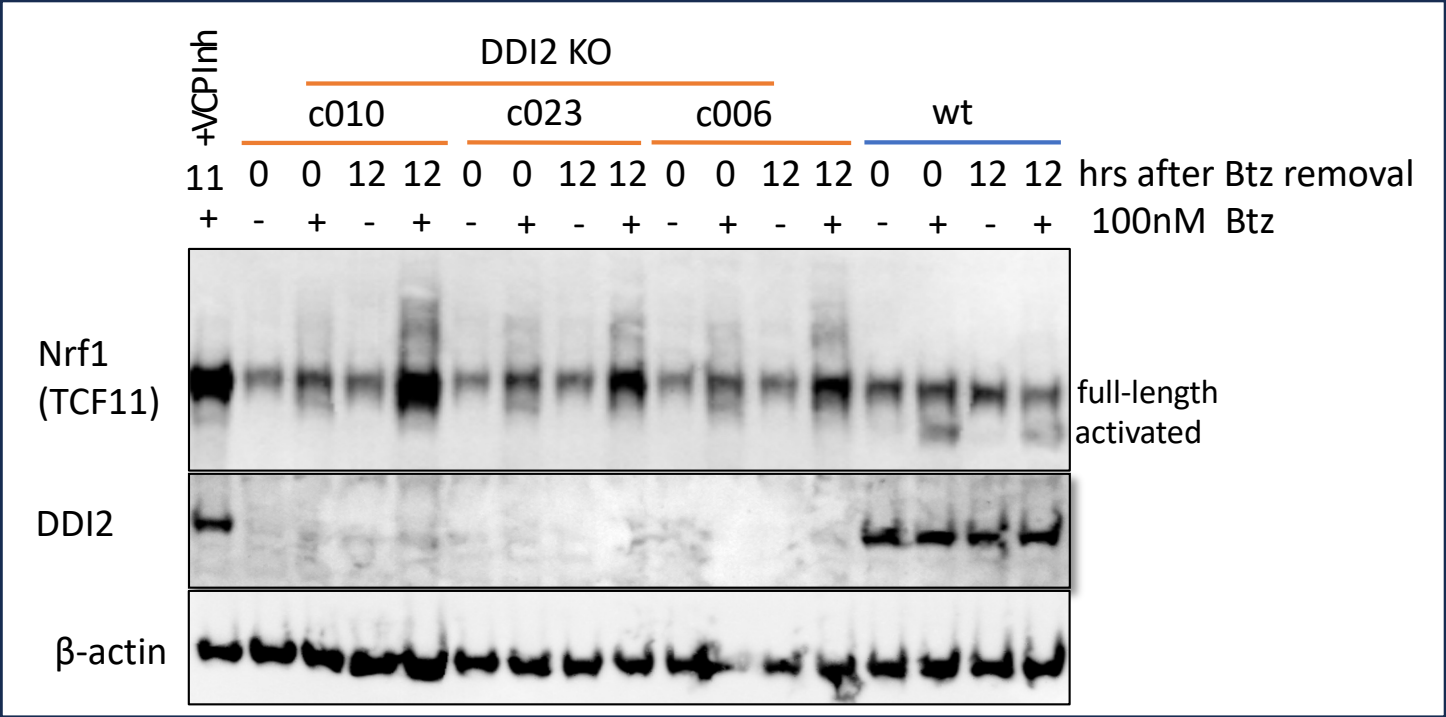

Nrf1 (TCF11)

DDI2 Ab after Nrf1 antibodies

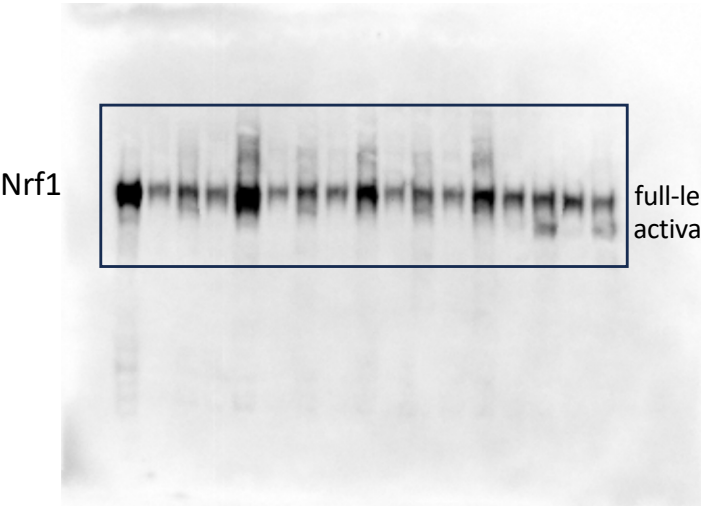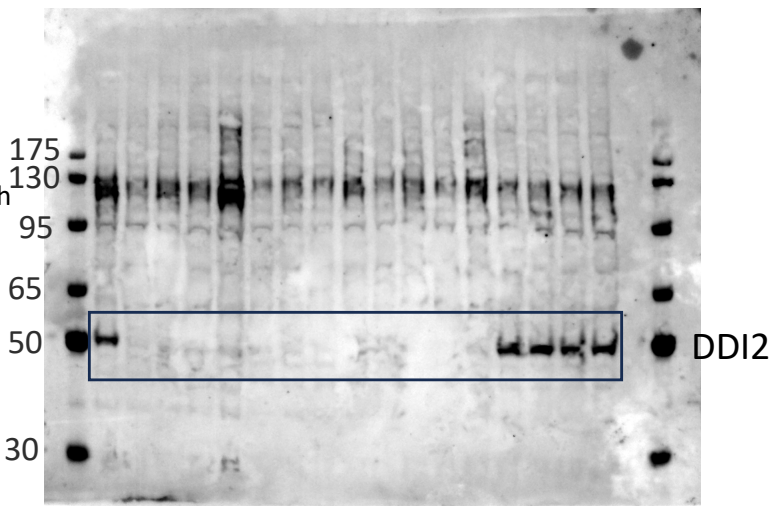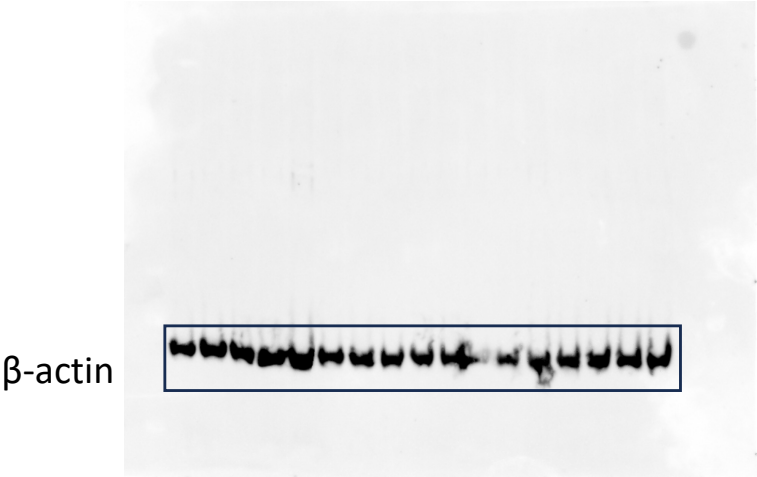

Supplement: Figure 1—source data 3. [file elife-91678-fig1-data3.zip › Fig1_source3.pdf]

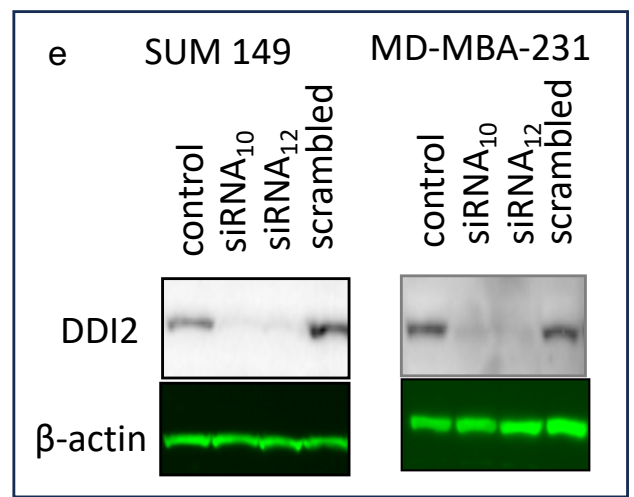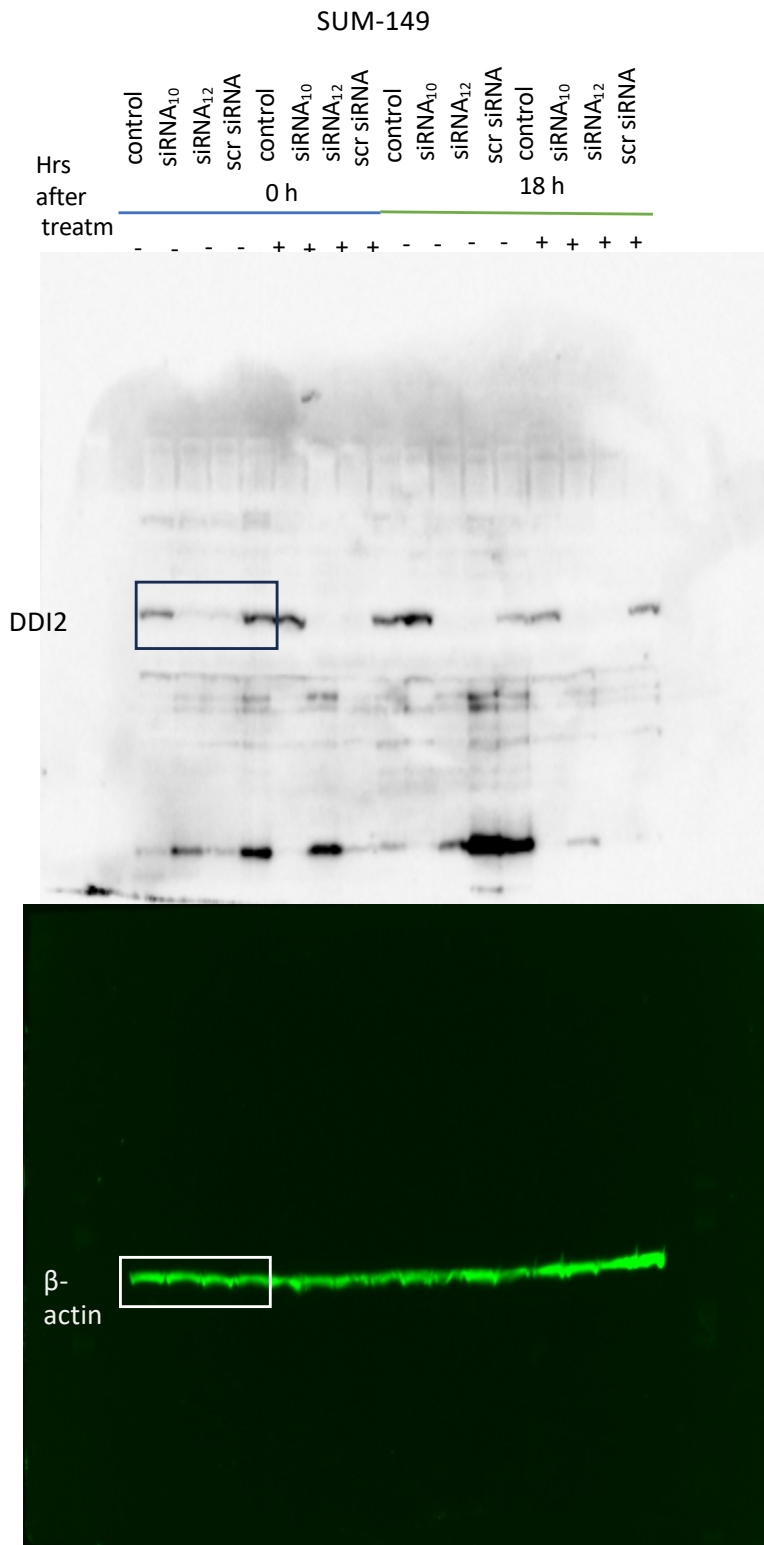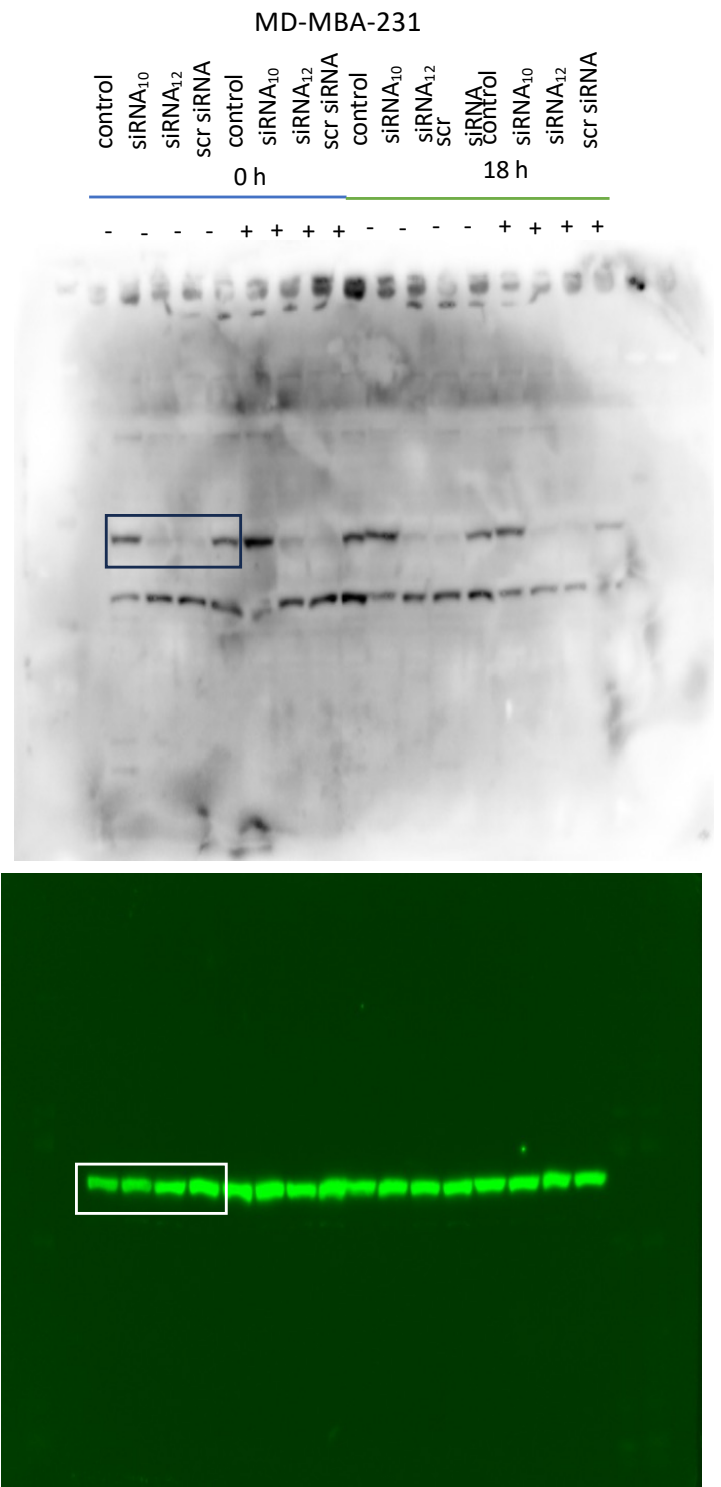

Supplement: Figure 1—source data 4. — Additional lanes demonstrate that the knockdown of DDI2 is maintained throughout the experiment. [file elife-91678-fig1-data4.zip › Fig1_source4.pdf]
