# Supplementary material for: Early recovery of proteasome activity in cells pulse-treated with proteasome inhibitors is independent of DDI2
Source: eLife. 2024 Apr 15;12:RP91678. doi: 10.7554/eLife.91678 (PMC11018354; doi:10.7554/eLife.91678)
Supplement: Supplementary file 1. [file elife-91678-supp1.docx]

**Supplementary Table.** **qPCR primers**.

| **Subunit** | **Gene** | **Primers** | **Sequences** | **Source** |
| --- | --- | --- | --- | --- |
| ⍺6 | PSMA1 | forward | AGAGCTTGCAGCTCATCAGA | PMID: 24998528 |
|  |  | reverse | CAAGACGAGACACAGGCAGT | PMID: 24998528 |
| ⍺2 | PSMA2 | forward | GCCCCGATTACAGAGTGC | PMID: **21911472** |
|  |  | reverse | TGGACGAACACCACCTGA | PMID: **21911472** |
| ⍺7 | PSMA3 | forward | GGTGCGCAACTCTACATGATTG | ThermoFisher Hs00138242_CE, A15629 |
|  |  | reverse | TCCTCTGCCTCGTATCAGTTATTAA | ThermoFisher Hs00138242_CE, A15630 |
| ⍺3 | PSMA4 | forward | CATTGGCTGGGATAAGCA | PMID: **21911472** |
|  |  | reverse | ATGCATGTGGCCTTCCAT | PMID: **21911472** |
| ⍺4 | PSMA7 | forward | CTGTGCTTTGGATGACAACG | PMID: **22932898** |
|  |  | reverse | CGATGTAGCGGGTGATGTACT | PMID: **22932898** |
| β7 | PSMB4 | forward | TCTCGGCCAGATGGTGAT | PMID: **29941490** |
|  |  | reverse | CACATAACCGAGGAAGCT | PMID: **29941490** |
| β5 | PSMB5 | forward | GCTTGCCAACATGGTGTATC | PMID: 24998528 |
|  |  | reverse | ATCATAGGCCTGCTCCACTT | PMID: 24998528 |
| β2 | PSMB6 | forward | CCAAGGAAGAGTGTCTGCAA | PMID: 24998528 |
|  |  | reverse | TGCATCAGTACAGGGCATCT | PMID: 24998528 |
| β1 | PSMB7 | forward | ATTGACCTCTGCGTCATCAG | PMID: 24998528 |
|  |  | reverse | CTGTTTCTTCCAGCACCTCA | PMID: 24998528 |
| Rpt2 | PSMC1 | forward | GATGACCTCTCTGGTGCTGA | PMID: 24998528 |
|  |  | reverse | CCCTTTCAGGGATTGAGAAA | PMID: 24998528 |
| Rpt5 | PSMC3 | forward | CCAGAATCATGCAGATCCAC | PMID: 24998528 |
|  |  | reverse | GCCTTCCATGTAGTCCTCGT | PMID: 24998528 |
| Rpt3 | PSMC4 | forward | CCGCCAGAAGAGATTGATTT | PMID: 24998528 |
|  |  | reverse | ACAATGTAGCGGTTTTCACG | PMID: 24998528 |
| Rpn2 | PSMD1 | forward | GGGGACCTCTTCAATGTCAA | PMID: **20385086** |
|  |  | reverse | TAGGCAGAGCCTCATTTGCT | PMID: **20385086** |
| Rpn10 | PSMD4 | forward | AGGAGGAGGCCCGGC | PMID: 22921402 |
|  |  | reverse | TCACTTCTTGTCTTCC | PMID: 22921402 |
| Rpn7 | PSMD6 | forward | CAGTCAGCTGCTGGAATCAT | PMID: 24998528 |
|  |  | reverse | TGGTACTGCCAGTTCTTGCT | PMID: 24998528 |
| Rpn6 | PSMD11 | forward | GCTGCTCTGGAAACAATTCA | PMID: 24998528 |
|  |  | reverse | ACAGATGCACCAAATGAGGA | PMID: 24998528 |
| Rpn5 | PSMD12 | forward | TTGGTCCACACTTGTTGAGG | PMID: 24998528 |
|  |  | reverse | TGAGAGAAAGGCTTCGGACT | PMID: 24998528 |
| Rpn11 | PSMD14 | forward | TTGGATGGAAGGTTTGACAC | PMID: 24998528 |
|  |  | reverse | CCACATGTTCCTCCAAATGA | PMID: 24998528 |
|  | \| PGK1 \|  \| \| --- \| --- \| | forward | AAAGTCAGCCATGTGAGCACT | PMID: 24998528 |
|  |  | reverse | CCACCCCAGGAAGGACTTTA | PMID: 24998528 |
